# The use of cognitive task analysis in clinical and health services research — a systematic review

**DOI:** 10.1186/s40814-022-01002-6

**Published:** 2022-03-08

**Authors:** Lizzie Swaby, Peiyao Shu, Daniel Hind, Katie Sutherland

**Affiliations:** 1 ScHARR, University of Sheffield, Sheffield, S1 4DA UK; 2 China National Petroleum Corporation (CNPC) Research Institute of Safety & Environment Technology, PetroChina Innovation Base, Shahe Town, Changping District, Beijing, China

**Keywords:** Cognitive task analysis, Medical decision-making, Clinical decision-making

## Abstract

**Background:**

At times, clinical case complexity and different types of uncertainty present challenges to less experienced clinicians or the naive application of clinical guidelines where this may not be appropriate. Cognitive task analysis (CTA) methods are used to elicit, document and transfer tacit knowledge about how experts make decisions.

**Methods:**

We conducted a methodological review to describe the use of CTA methods in understanding expert clinical decision-making. We searched MEDLINE, EMBASE and PsycINFO from inception to 2019 for primary research studies which described the use of CTA methods to understand how qualified clinicians made clinical decisions in real-world clinical settings.

**Results:**

We included 81 articles (80 unique studies) from 13 countries, published from 1993 to 2019, most commonly from surgical and critical care settings. The most common aims were to understand expert decision-making in particular clinical scenarios, using expert decision-making in the development of training programmes, understanding whether decision support tools were warranted and understanding procedural variability and error identification or reduction. Critical decision method (CDM) and CTA interviews were most frequently used, with hierarchical task analysis, task knowledge structures, think-aloud protocols and other methods less commonly used. Studies used interviews, observation, think-aloud exercises, surveys, focus groups and a range of more CTA-specific methodologies such as the systematic human error reduction and prediction approach. Researchers used CTA methods to investigate routine/typical (*n* = 64), challenging (*n* = 13) or more uncommon, rare events and anomalies (*n* = 3).

**Conclusions:**

In conclusion, the elicitation of expert tacit knowledge using CTA has seen increasing use in clinical specialties working under challenging time pressures, complexity and uncertainty. CTA methods have great potential in the development, refinement, modification or adaptation of complex interventions, clinical protocols and practice guidelines.

**Registration:**

PROSPERO ID CRD42019128418.

**Supplementary Information:**

The online version contains supplementary material available at 10.1186/s40814-022-01002-6.

## Background

Decision-making is ubiquitous in clinical practice, as health professionals gather information; evaluate test results; define problems; set treatment goals; start, stop or delay treatment; and advise, refer, admit or discharge patients [1]. Decisions are affected by factors such as their difficulty and familiarity [2]. People have a limited capacity for processing information [3], which makes it increasingly difficult to adequately understand a decision situation and the range of possible actions, the greater the levels and types of uncertainty involved [4, 5]. Any adequate account of clinical decision-making must also deal with how clinicians handle uncertainty, which is also pervasive in medicine [6, 7].

How we recognise, classify and reduce uncertainty is the subject of a vast literature [8, 9] with poor information, inadequate understanding, indeterminacy, complexity, ambiguity, unpredictability of phenomena, conflicting rules and beliefs affecting our grasp on situations and outcomes [8, 10–12]. Decision support algorithms often need to be used in combination with other methods of inference [13]; in the absence of tractable problems and shared assumptions about phenomena that are well-defined and relatively objective, such deductive approaches to problem-solving may perform poorly [14–17]. The theory of bounded rationality predicts that under our computational, environmental and epistemological constraints, we become satisficers, drawing on heuristics to inductively infer optimal, rather than deductively establishing perfect, solutions [18, 19]. In such situations, the tacit knowledge of clinicians — the sort that is not easily defined or learned [20] — is critical as they attempt to synthesise patient values and best, if often still ambiguous, research evidence [21].

As technological advances increase cognitive demands on people [22], it becomes more important to incorporate the cognitive aspects of performance into task protocols and systems in which they make inferences, diagnoses, judgements and decisions [23]. The increasing complexity of healthcare systems is a challenge for health professionals and researchers and a risk for patients [24–27].

How clinicians use tacit knowledge to make decisions under conditions of uncertainty has been flagged as critical for the development of interventions in areas including emergency medicine [28], prescribing [29], mental health [30], liver cirrhosis [31], urology [32] and the management of care transitions [33]. The new Medical Research Council (MRC) framework calls for the use of novel designs that can help reduce decision-maker uncertainty and to assess the feasibility of interventions through establishing optimal content and delivery [34]. Intervention development requires that one properly formulates a problem, determines needs, examines current practice and context, and models process and outcomes, all of which inform the feasibility of an intervention [35]. Cognitive task analysis is an umbrella term for tools and techniques used in describing the knowledge and strategies that are used in making judgements about situations and goals and making decisions. One objective of CTA methods, and the naturalistic decision-making paradigm from which they derive, is to help experts to express tacit knowledge, enabling researchers and novices to learn and systems to be improved [36]. This makes CTA methods useful in assessing the feasibility and usability of interventions [37–41].

Despite an increasing concern with decision-making, uncertainty and complexity in the health science literature, the utility of CTA methods has received little attention. Their use in clinical decision-making has not been the subject of a systematic overview. They are not amongst the methods discussed by the MRC framework as approaches to developing and evaluating complex interventions [34, 42, 43], although complex interventions often involve decision-making [44–47], and, in an editorial in this journal, Pat Hoddinott flags the roles of tacit knowledge and fast-and-slow thinking in intervention development [45]. For these reasons, we undertook a systematic methodological review [48] to understand how CTA methods are being used in real-world clinical settings and which objectives others have deemed them useful to address.

## Methods

This review was registered on the PROSPERO database on 15 April 2019, ID CRD42019128418 [49], and has been conducted and reported according to PRISMA guidelines [50]. Published primary research studies were eligible if they (1) described the use of CTA methods to understand how (2) qualified clinicians (3) made clinical decisions (4) in real-world clinical settings. Studies were ineligible if (1) they did not use CTA methods; (2) the participants were students, patients or members of the public; (3) there was no decision (just a simple task breakdown) or decisions were non-clinical; or (4) the setting was simulated, rather than a real-world environment. Objectives of studies concerning non-clinical decisions that were excluded are improvement of the physical environment [51], assessing the usability of information technology [52] and workplace modelling [53]. Studies which used CTA methods to test an already developed simulator were excluded; studies which used them to gather data about real-world environments to develop a simulator were included. Conference abstracts, and unpublished literature, were included where eligible. There were no date restrictions on when articles were published, and articles in any language were included. Systematic reviews, evidence-based guidelines, literature reviews, commentaries and opinion pieces were excluded.

An initial MEDLINE scoping search in December 2018 identified that Medical Subject Headings (MeSH terms) were overly sensitive in their retrieval of relevant articles. Screening a random sample of 100 out of 29,380 citations indexed with “task performance and analysis” — the MeSH term most commonly associated with CTA studies in our scoping search, we found no CTA studies. Therefore, we took the unusual step of using only free-text terms in the searches — those which described CTA methods and related terms, e.g. “Applied Cognitive Task Analysis” and “Critical Decision Method” [54]. The full search strategy is outlined elsewhere [49].

The search terms were applied in MEDLINE, EMBASE and PsycINFO, through Ovid. Searches covered studies from database inception (MEDLINE 1966, EMBASE 1947, PsycINFO 1967) to 12th March 2019. Where articles could not be retrieved through copyright libraries, the reviewers contacted authors directly to obtain copies. Four potentially eligible articles were excluded as we were unable to retrieve the full text. Of the remainder, duplicates were removed, and two reviewers used the predefined eligibility criteria to identify eligible citations, working independently and in duplicate. Full documents were retrieved, and assessed for eligibility, with reasons for rejection documented. A third reviewer resolved any disputes.

Descriptive data extraction tables were piloted before use by the two main reviewers. We extracted data into tables to include the following: country of study; clinical setting; study aims/objectives; clinical specialty; CTA methods used [54]; data capture method (interviews, self-reports, observation, automated capture); data targets (past, present or future); whether events described were routine/typical, challenging or rare events/anomalous; generality of events (e.g. job/task, abstract/general or incident/event); and data presentation (e.g. textual descriptions, tables, graphs) [54]. Once data extraction was complete, tables were used to group some of the columns for presentation of results, and filters were applied to summarise counts for each column.

As summary effect measures were not the primary goal of this methodological study, we did not collect data on, or assess, risk of bias either for individual studies or across studies [48, 55].

## Results

### Summary of included studies

**Fig. 1:**
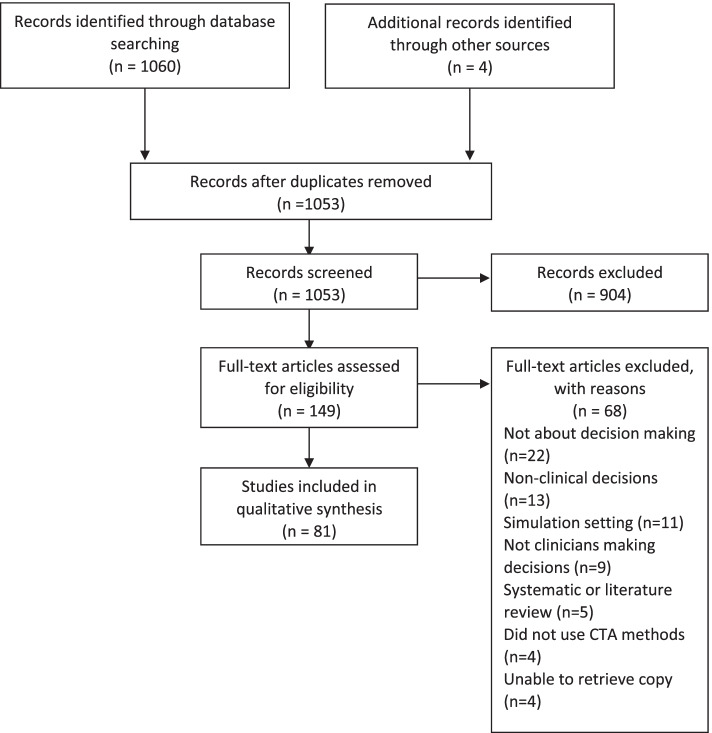
PRISMA flowchart for studies included

Electronic database searches retrieved 1060 results; a further four were identified through contact with an author to retrieve a full copy and through incidental identification during the data extraction phase. After duplicates were removed, 1053 articles remained, of which 904 were excluded at title and abstract stage. One-hundred and forty-nine articles were assessed at full text, and a further 68 were excluded, leaving 81 articles, representing 80 unique studies for inclusion in the review (Fig. 1).

Sixty-eight full-text articles were rejected because they were not looking at decision-making. Instead, these articles had focuses such as developing a description of steps for a procedure/task (*n* = 22); decisions were non-clinical (*n* = 13); they took place in a simulation setting (*n* = 11); decisions were being made by patients, the public or students rather than expert clinicians (*n* = 9); systematic or literature reviews with no primary research (*n* = 5); or they did not report on CTA methods being used (*n* = 4). In addition, copies of 4 articles could not be retrieved after contact with authors.

The five systematic or literature reviews that were excluded did not have a scope that overlapped with the scope of this review. These either focused on training or a specific procedure or setting.

**Fig. 2:**
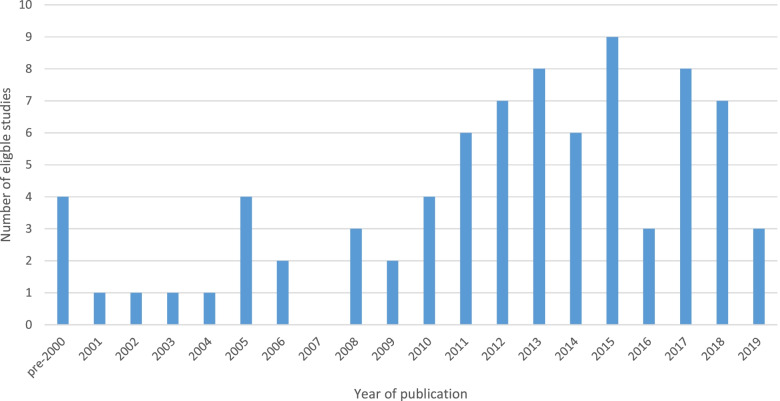
Number of studies using CTA methods by year (*n* = 80*). *Year of publication unknown for one publication. This was a conference poster identified through contact with an author to obtain a copy of a different article

There was an apparent increase in the numbers of published studies using CTA methods to understand expert clinical decision-making between 2006 and 2015 (Fig. 2). The earliest included study was published in 1993 and the most recent in 2019. The majority of studies were carried out in the USA (*n* = 48), with other countries of origin being the UK (*n* = 9); Canada (*n* = 5); Australia (*n* = 5); Slovakia (*n* = 3); Ireland (*n* = 2); and Taiwan (*n* = 2), and with one study carried out in each of Germany, Iran, France, Norway, Sweden and the Netherlands. CTA studies were carried out in hospitals (*n* = 60), university or medical schools (*n* = 11); pre-hospital settings (*n* = 3); the community (*n* = 5); and a military training command setting (*n* = 1). Clinical specialties represented were surgery (*n* = 30); critical or intensive care, including neonatal and paediatric settings, (*n* = 11); anaesthesia/trauma medicine (*n* = 10); general medicine (*n* = 7); pre-hospital and emergency medicine (*n* = 6); primary care (*n* = 6); infectious diseases (*n* = 4); obstetrics and gynaecology (*n* = 3); paediatrics (*n* = 2); interventional radiology (*n* = 2); neuro-rehabilitation (*n* =2); pathology (*n* = 1); pharmacy (*n* = 1); and diabetes education (*n* = 1).

### Aims and objectives of included studies

In a large number of studies, the primary aim was to use CTA to understand expert decision-making for management of a particular clinical scenario (*n* = 35). Other common objectives included using clinician decision-making in the development of training models or educational frameworks (*n* = 14); understanding management of a procedure to investigate whether a support tool or application is warranted (*n* = 7); investigating information omission when describing or teaching a procedure (*n* = 6); creating a framework to compare variability in a procedure (*n* = 4); error identification or reduction (*n* = 4); and comparing differences between novices and experts (*n* = 4). In smaller numbers of studies, objectives included understanding a procedure with the aim of optimisation of that procedure (*n* = 3); understanding critical steps in a procedure to provide effective teaching and procedure assessment (*n* = 3); breaking down steps in a task to understand key decision points (*n* = 2); developing a simulator (*n* = 1); a methodological investigation looking at how much critical information is gained from each CTA interview over and above information in a gold standard (*n* = 1); comparing two methods of undertaking the same procedure (*n* = 1); and determining the number of experts needed to develop gold standard protocols for a procedure (*n* = 1).

### Knowledge elicitation

There are a number of methods of knowledge elicitation described within CTA, as defined in Crandall et al. [54]. Of these methods, most studies included in this review used either critical decision method (CDM) (*n* = 36) or CTA interviews (*n* = 30) or both of these (*n* = 1). Less commonly used CTA methods were the following: hierarchical task analysis (*n* = 11); task knowledge structures (*n* = 8), which is a breakdown of task elements including relations between objects and associated actions and how they are represented in an individual’s memory [56]; think-aloud exercises (*n* = 4); team knowledge audit (*n* = 2); timeline analysis (*n* = 2); Systematic Human Error Reduction and Prediction Approach (SHERPA) (*n* = 2); and the concepts, processes and principles approach (*n* = 2). Some lesser known, less easily accessed methods which were seen in only one study each, included Patient and Community Engagement Research (PaCER) approach [57], task diagram construction [23], video timing analysis [58], distributed situation awareness [59], incident analysis [60], knowledge analysis [61] and Delphi method for consensus [62].

### CTA methods

In the 35 studies which had the aim of understanding expert decision-making in the management of a particular clinical scenario, most used cognitive task analysis interviews (*n* = 31), often using the critical decision method (*n* = 23). Other studies aimed to develop a training model or educational framework (*n* = 14) or to create a framework to compare variability in a procedure (*n* = 4). Studies with these aims, in addition to using cognitive task analysis interviews (*n* = 12), also used decomposition approaches such as task knowledge structures (*n* = 4) and hierarchical task analysis (*n* = 4), which are seen less often when the main aim is to understand expert decision-making in the management of a particular clinical scenario.

Most studies (*n* = 42) used a combination of data collection sources. Data were collected through interviews (*n* = 73), observation (*n* = 27), think-aloud exercises (*n* = 10), surveys and questionnaires (*n* = 6), focus groups (*n* = 5), review of protocols generated through interviews (*n* = 4) and SHERPA (*n* = 3). Additional, less commonly used methods of data collection were comparison of CTA interviews against a gold standard for a specific procedure (*n* = 2). consensus exercises (*n* = 2). self-reports (*n* = 2), automated capture (*n* = 2), task breakdown (*n* = 1) and generation of a description whilst simulating a procedure (*n* = 1).

### Data targets and focus of included studies

Data targets, or the focus of the decision-making investigation, were mostly based in the past (*n* = 41) and in the present (*n* = 36), with a small number based in the future (*n* = 2), and insufficient information in one article. Studies using data targets in the past often tended to do so by asking clinicians to recall a specific case or incident [63, 64]. Those studies that used data targets in the present tended to use interviews, observations and think-aloud exercises to look at procedures and practice either in real time, or shortly after the event, using specific cases or think-aloud for a theoretical process. For example, one study used interviews and think-aloud exercises to identify task cues in critical combat medical procedures [65], whilst another used interviews to understand principles guiding clinicians’ decisions whilst undertaking surgical procedures [66]. Future data targets included participants being presented with a clinical scenario of symptomatic gallstones and being interviewed on the decisions they would make for this patient [67]. The events investigated in eligible studies were largely routine/typical events (*n* = 64), with fewer studies investigating challenging (*n* = 13) or more uncommon, rare events and anomalies (*n* = 3).

Articles were predominantly focussed on either an overall job or task (*n* = 37), such as investigating the clinical knowledge omitted when teaching cricothyrotomy and focussing on the procedure itself rather than specific patient cases [68], or specific incidents or events (*n* = 35) such as describing cues used by nurses in decision-making when responding to a clinical alarm, involving recall of individual cases [69]. There were a smaller number of studies that looked at more abstract or general tasks (*n* = 7). These investigated more general settings such as identifying decisions, cues and novice traps in laparoscopic surgery [70]; understanding how emergency physicians and residents experience busy emergency department environments [71]; and understanding the cognitive and physical processes of interventional radiology when eliciting relevant information for user interface design for human-computer interaction [72]. The level of task generality was unclear in one article.

### Presentation of results

In addition to textual descriptions as a method of displaying results, most articles used tables, graphs and/or illustrations (*n* = 66), with others using qualitative models such as flowcharts (*n* = 30) and simulation; numerical and symbolic models (*n* = 4), such as task timelines, an objective scale for assessment of technical skills (OSATS) scale; or a taxonomy containing job, task, subtask, element and motion levels. In addition, some articles used hierarchical task analysis decomposition trees, a tree-like diagram breaking down steps and substeps in a procedure, for example, laparoscopic cholecystectomy [73]. Others used workflow models; a step by step instructional guide with decision points and “if” statements [74], decision analysis tables, such as a breakdown of decision points and how these relate to action, cause and outcome for each situation [75] and procedural maps; a mapping diagram linking steps in a straightforward procedure, steps that may change from the original plan, and other steps that can affect a decision whilst undertaking a procedure, such as laparoscopic cholecystectomy [76].

## Discussion

This review shows a steady increase in the use of CTA methods in health research over 20 years, with applications such as transferring expert knowledge to novices and providing the decisional architecture for simulators, education or training modules. CTA is more commonly used in surgery and intensive care research and least frequently in pharmacy and interventional radiology. This perhaps reflects an abiding concern with the time pressures, complexity and uncertainty that surround decision-making in these contexts [77–82]. The most commonly used CTA methods were interviews, especially using CDM, and slightly less frequently hierarchical task analysis (HTA). There are a number of CTA methods for which no published application was found, although this might reflect the scope of the review. For instance, many HTA studies focused narrowly on task breakdown, without extending analysis to an understanding of decision-making.

This is the largest overview of the use of CTA in clinical and healthcare settings. It expands considerably on a previous review of seven nursing studies, which found CDM a valuable tool for eliciting expert knowledge [83]. We consider a sample of 80 studies adequate to characterise approaches to the use of CTA in health sciences that resort to further databases, and the grey literature would yield diminishing marginal returns [84].

In verbal accounts, experts may leave out up to 70% of components and decision steps needed to successfully perform a task, resulting in intervention protocols that are incomplete or ineffective [68]. That experts cannot themselves articulate what they do, together with optimism bias and groupthink [45], may explain the generally disappointing performance of theory-based [85] and manualised [86] interventions in healthcare and the abiding concern with better specification of interventions [87–89]. It is often undesirable to standardise complex interventions [42, 90]. But amid complaints about “cookbook” approaches [91], even the greatest advocates of flexible delivery acknowledge the need for a stable core [92], without which interventions cannot be differentiated [93, 94] or best practice defined and implemented. The current trend in health sciences is a more sophisticated approach to standardisation. Surgeons advocate detailed task decomposition, descriptions of the conditions and limits to standardisation [95] and that intervention stability is assessed before clinical outcomes [96]. Mental health researchers recognise that contingencies require documented adaptations to evidence-based practices [97]. As this more nuanced style of documentation is dependent on integrating best evidence with tacit knowledge [98], CTA methods are ideally placed to support the development, standardisation and adaptation of complex interventions — as well as training in their application. Several of the included studies also show the potential of CTA methods in the creation and modification of other care process specification documents — practice guidelines, decision rules, pathways, care plans and care maps [99]. In the domain of quality improvement, this review provides examples of the use of the HTA and SHERPA approaches for error identification/reduction, CTA interviews and HTA for understanding variation in practice, particularly within multidisciplinary teams [100]. These methods may have application in clinical areas where reducing variation in practice is seen as desirable [101]. CTA methods seem well-suited to elicit, document and share the tacit knowledge used in decision-making. Meta-analyses comparing CTA with other training methods have identified large effects in terms of procedural knowledge and technical performance [102, 103].

The scope of this review did not include studies that primarily focused on simulation studies or those in educational or training settings. We excluded studies focused on the use of CTA methods to evaluate simulations or educational programmes, unless part of their remit also included the decision-making of qualified health professionals in naturalistic settings. During the selection process, we excluded large numbers of studies evaluating simulations, indicating the potential for a systematic overview. Sound meta-analytic evidence already exists for the effectiveness of CTA as a basis for training, compared with other approaches [103], as well as smaller systematic reviews on its application in surgical education [104, 105]. As it is more intensive, a cost-utility analysis, modelling the incremental cost of improved learning outcomes and subsequent performance, may be required to introduce it into practice.

### Limitations

This review was purely descriptive, did not assess outcomes of included studies and risk of bias was not assessed. Some readers will feel that reviews must always be evaluative, with endpoints and critical appraisal a necessary condition, but our approach is in line with guidance on methodological reviews [48, 55] and the conduct of many, including in this journal [106–108]. Individuals often overestimate their own expertise or vary in their self-assessment according to context [109, 110]. Further research on this topic could assess expertise using evidence of qualifications, track record, experience and competency in applying knowledge and experience to new situations, although these approaches may not be applicable to all forms of expert knowledge [111, 112]. The use of CTA methods appears to have increased over recent years, before a small decrease. It has been reported that there is a sustained interest in CTA methods in relation to training [102], but the number of studies eligible for our study (Fig. 2) is too small to rule out chance variation. The sciences and social sciences represent diverse, competing schools of thought and methodological approaches, and it is common that promising approaches do not become permanently embedded in routine practice [113–116].

Furthermore, we were unable to retrieve four apparently eligible articles through copyright libraries or attempts to contact authors. On the basis of the titles and abstracts, these articles [117–120] replicate settings (dental hygiene, endoscopic surgery) and applications (elicitation of expert knowledge, task description) documented in other studies. We took a liberal view of expertise as characteristic of qualified — as opposed to unqualified — health professionals. In applying CTA methods, researchers may consider circumscribing specialist subgroups of qualified clinicians, based on peer nomination, influence and cues such as certification, past performance or test results [121].

## Conclusions

The use of CTA methods appears to be increasing in clinical specialties characterised by time scarcity, complexity and uncertainty. However, numbers are small, and this review only looked at CTA methods used in expert clinical decision-making. There are many other applications of CTA not covered in this review, such as novice decision-making, training and education. Whilst the role of CTA methods in education is well documented, they have under-recognised potential in the development, refinement, modification or adaptation of complex interventions and care process specifications.

## Supplementary Information

**Additional file 1: Supplementary Table 1.** Reasons for exclusion of articles at full text.

**Additional file 2: Supplementary Table 2.** Data extraction for eligible studies.

## Data Availability

Not applicable.
