# Supplementary material for: The use of cognitive task analysis in clinical and health services research — a systematic review
Source: Pilot Feasibility Stud. 2022 Mar 8;8:57. doi: 10.1186/s40814-022-01002-6 (PMC8903544; doi:10.1186/s40814-022-01002-6)
Supplement: Supplementary file 1 — Additional file 1: Supplementary Table 1. Reasons for exclusion of articles at full text. [file 40814_2022_1002_MOESM1_ESM.docx]

**Supplementary table 1: Reasons for exclusion of articles at full text**

| **Author** | **Title** | **Rationale for exclusion** | **Summary exclusion** |
| --- | --- | --- | --- |
| Al-Hakim (2014) | Applying hierarchical task analysis to improving the patient positioning for direct lateral interbody fusion in spinal surgery | Task description for patient positioning | Not about decision making |
| Al-Hakim (2015) | Human error identification for laparoscopic surgery: Development of a motion economy perspective | Not CTA, observation and development of taxonomy | Not about decision making |
| Armstrong (2012) | Standardization of surgical procedures for identifying best practices and training | Developing a taxonomy (description of steps) of a procedure | Not about decision making |
| Barber (unpublished) | Scaling up: a principled approach for primary care transformation in Alberta | not relevant for our topic – a principled approach for primary care transformation in Alberta | Not clinicians making decisions |
| Barnard (2004) | Bridging between basic theory and clinical practice | Review article/summary of processes | Systematic or literature review |
| Beuscart-Zephir (2010) | Example of a Human Factors Engineering approach to a  medication administration work system: Potential impact on patient safety | Observation/document review, then interviews with nurses to comment on processes in the interpretation – software models to model tasks observed | Simulation setting |
| Bhattacharyya (2017) | A Randomized Controlled Trial Evaluating the Effectiveness of the Imperial Knee Arthroscopy Cognitive Task Analysis (IKACTA) Tool | Evaluating a CTA tool | Simulation setting |
| Bhattacharyya (2018) | Trauma simulation training: a randomized controlled trial evaluating the effectiveness of the Imperial Femoral Intramedullary Nailing  Cognitive Task Analysis (IFINCTA) tool | Evaluation of a CTA tool by undergraduates/novices | Simulation setting |
| Blandford (2004) | Situation awareness in emergency medical dispatch | Ambulance controllers – perform/decisions in major incidents | Not clinicians making decisions |
| Brannon (2006) | Ad Hoc versus Standardized Admixtures for Continuous Infusion Drugs in Neonatal Intensive Care: Cognitive Task Analysis of Safety at the Bedside | Not CTA | Did not use CTA methods |
| Breen (2014) | Defining a competency  map for a practical skill | Focus groups with experts to do HTA of spinal anaesthesia, mapping software used to present information, Software developed as bedside assessment tool to link with HTA. Not clinical decision making. | Not clinicians making decisions |
| Cameron (2000) | A cognitive task analysis for dental hygiene | Unable to retrieve copy | Unable to retrieve copy |
| Chana (2017) | Improving specialist drug prescribing in primary care using task and error analysis:  an observational study | Interviews and observations looking at prescribing errors to see if clinical decision support system is needed for GPs, not CTA, just how could system support task | Non-clinical decisions |
| Cioffi (2012) | Expanding the scope of decision-making research for nursing and midwifery practice | Review article of CDM approaches to research | Systematic or literature review |
| Colligan (2010) | Does the process map influence the outcome of  quality improvement work? A comparison of a sequential flow diagram and a hierarchical task analysis diagram | Whether different representations of a process affect perceptions of risk | Did not use CTA methods |
| Colligan (2012) | Designing for distractions: a human factors approach to decreasing interruptions at a centralised medication station | Breakdown of tasks to develop intervention, then testing that intervention | Not about decision making |
| De Vries (2018) | Development and validation of the TOCO–TURBT tool: a summative assessment tool that measures surgical competency in transurethral  resection of bladder tumour | Evaluation of a CTA developed tool, doesn’t report development of tool | Simulation setting |
| Demirel (2016) | A Hierarchical Task Analysis of Cricothyroidotomy Procedure for a Virtual Airway Skills Trainer (VAST) Simulator | Looking at performance, scoring tasks for forthcoming VR simulator | Not about decision making |
| Demirel (2017) | A hierarchical task analysis of shoulder arthroscopy for a virtual arthroscopic tear diagnosis and evaluation platform (VATDEP) | Looking at performance, scoring tasks for forthcoming VR simulator | Not about decision making |
| Dionne-Odom (2015) | Conceptualizing surrogate decision making at end of life  in the intensive care unit using cognitive task analysis | CTA but for surrogate decision making | Not clinicians making decisions |
| Dionne-Odom (2014) | A Theoretical Model of the Psychological Processes of Surrogate Decision Making at Adult End of Life in the ICU Using Cognitive Task Analysis | CTA interviews but for surrogate decision making | Not clinicians making decisions |
| Dominguez (1995) | Perception-action coupling in endoscopic surgery: A cognitive-task analysis approach | Unable to retrieve copy | Unable to retrieve copy |
| Embrey (2013) | The use of cognitive task analysis to capture expertise for tracheal extubation training in anaesthesiology | CTA based instruction vs non-CTA in training anaesthesia staff | Non-clinical decisions |
| Faiella (2018) | Expanding healthcare failure mode and effect analysis: A composite proactive risk analysis approach | Not CTA, risk reduction | Not clinicians making decisions |
| Fernandez-Gutierrez (2015) | Comparative ergonomic workflow and user experience analysis of MRI versus fluoroscopy-guided vascular interventions: an iliac angioplasty exemplar case study | Comparing standard protocol vs MRI guided, simulation | Simulation setting |
| Foster (2016) | Application of objective clinical human reliability analysis (OCHRA) in assessment of technical performance in laparoscopic  rectal cancer surgery | No CTA, task breakdown analysis to describe laparoscopic TME | Not about decision making |
| Fruggiero (2018) | What humans act in robotic surgery | Evaluating robotic surgery and interaction with humans, not CTA | Simulation setting |
| Funk (2010) | Use of modeling to identify vulnerabilities to human error in laparoscopy | Unable to retrieve copy | Unable to retrieve copy |
| Gauthier (2009) | Validating and representing case based knowledge | Representing knowledge for education | Non-clinical decisions |
| Geis (2018) | A Validation argument for a simulation-based training course centered on Assessment, Recognition and Early Management of Pediatric Sepsis | Simulations for trainees (CTA not used to develop simulation) | Simulation setting |
| Ghasemi (2015) | Identification and assessment of medical errors in the triage area of an educational hospital using  the SHERPA technique in Iran | SHERPA to identify errors and risks, observation, interviews, guideline review, not CTA | Did not use CTA methods |
| Gucev (2013) | Cognitive task analysis for instruction in single-injection ultrasound guided-regional anaesthesia | CTA interviews with experts to elicit knowledge for instructing novice learners in US guided regional anaesthesia, doesn’t report results of CTA work with experts | Not clinicians making decisions |
| Hegde (2018) | A cognitive task analysis approach toward identifying learning requirements and informing training design for different categories of learners of endoscopic submucosal dissection | Conference abstract – simulation vs non simulation | Simulation setting |
| Huang (2014) | Systematic engineering tools for describing and improving medication administration processes at rural healthcare facilities | Description of tasks, not trying to understand decisions | Not about decision making |
| Hysong (2010) | Provider management strategies of abnormal test  result alerts: a cognitive task analysis | CTA interviews on how managed alerts in a system. Not clinical decision making | Non-clinical decisions |
| Klein (1999) | Features of problem detection | Review, but does some interviews, looking at threshold for problem detection, not just medical | Systematic or literature review |
| Koopman (2015) | Physician Information Needs and Electronic Health  Records (EHRs): Time to Reengineer the Clinic  Note | CTA on how notes are viewed when preparing for clinic visits, to try and improve display of EHR | Non-clinical decisions |
| Kushniruk (2001) | Analysis of Complex Decision-Making Processes in Health Care: Cognitive Approaches to Health Informatics | Review article | Systematic or literature review |
| Kyaw (2012) | Simulating various levels of clinical challenge in the assessment of clinical procedure competence | Comparing performance with simulator between novice/expert | Simulation setting |
| Lane (2006) | Applying hierarchical task analysis to medication administration errors | HTA to demonstrate types of errors and where in process (drug administration) | Non-clinical decisions |
| Luko (2017) | Cognitive task analysis of spatial skills in hysterectomy with the Da Vinci surgical system | Looking at task breakdown and insight into spatial demands during robot assisted surgery | Not about decision making |
| Madani (2018) | Measuring Decision-Making During Thyroidectomy: Validity Evidence for a Web-Based Assessment Tool | Educational platform with built in live feedback on performance | Non-clinical decisions |
| Madani (2017) | Measuring intra-operative decision-making during laparoscopic cholecystectomy: validity evidence for a novel interactive Web-based assessment tool | Testing an e-learning platform | Non-clinical decisions |
| Malhotra (2005) | Designing the design phase of critical care devices: a cognitive approach | Not CTA | Did not use CTA methods |
| Mazloumi (2018) | Customization and validation study of WHO surgical safety checklist as a tool to control medical error in operation rooms in Iran | Cross-country validation of a surgical checklist rather than studying naturalistic decision making | Non-clinical decisions |
| Nemani (2014) | A Comparison of NOTES Transvaginal and Laparoscopic Cholecystectomy Procedures Based upon Task Analysis | Looking at tasks and subtasks within a procedure to help with training | Not about decision making |
| Nemani (2013) | Hierarchical task analysis of hybrid rigid scope Natural Orifice Translumenal Endoscopic Surgery (NOTES) cholecystectomy procedures | Author advised this was a conference version of the above peer reviewed paper. Not included. | Systematic or literature review |
| O’Donnell (2011) | Development of an optimal patient transfer task set and simulation-based intervention to reduce musculoskeletal injury in healthcare workers | HTA to develop simulation to help future trainees | Non-clinical decisions |
| Parameshwara (2016) | NGOMSL Simulation model in an emergency department | Looking at observation of tasks completed in a shift and time taken to do | Non-clinical decisions |
| Peyre (2008) | Advanced surgical skill assessment: task analysis as model for the development of a valid and reliable objective procedural checklist | Description of tasks to create an instrument and rate it | Not about decision making |
| Phipps (2008) | Human factors in anaesthetic practice: insights from a task analysis | Task breakdown then risk identification at each step, suggestions for future research | Not about decision making |
| Plumptre (2017) | Standardizing bimanual vaginal examination using cognitive task analysis | Expert interviews to evaluate a model for use in teaching students | Simulation setting |
| Porat (2016) | Eliciting User Decision Requirements for Designing  Computerized Diagnostic Support for Family  Physicians | Does not report on CTA for understanding, interviews used to answer questions on how a decision support computer tool could help avoid errors | Non-clinical decisions |
| Potworowski (2016) | Training change agents in CTA to bring health care transformation to scale: The case of primary care practice facilitators | looking at organisational approach, not patient level decision making | Not clinicians making decisions |
| Pusic (2001) | Design of computer-aided instruction for radiology interpretation: the role of cognitive task analysis | Describes cognitive task analysis model for students, no experts, no data | Non-clinical decisions |
| Sarker (2006) | Self-appraisal hierarchical task analysis of laparoscopic surgery performed by expert surgeons | Appraisal of videos of experts performing procedure | Not about decision making |
| Sarker (2008) | Procedural performance in gastrointestinal endoscopy: An assessment and self-appraisal tool | Observation and scoring of a procedure by novices/experts | Not about decision making |
| Sarker (2008) | Constructing hierarchical task analysis in surgery | HTA description of tasks/subtasks for surgical procedure by observation | Not about decision making |
| Sarker (2006) | Development of assessing generic and specific technical skills in laparoscopic surgery | Develop assessment tool to assess technical skills in lap surgery, HTA to breakdown tasks, scored by experts – tool is for appraisal of surgical technique | Not about decision making |
| Sarker (2010) | Assessing Operative Performance in Advanced Laparoscopic Colorectal Surgery | HTA for laparoscopic colectomy, procedures scored by experts, appraisal of ability between novice/expert | Not about decision making |
| Suebnukarn (2015) | Understanding information synthesis in oral surgery for the design of systems for clinical information technology | Dental students decision making in pre-operative scenarios | Not clinicians making decisions |
| Sreeramakavacham (2018) | Effect of Patient Acuity of Illness and Nurse Experience on EMR Works in Intensive Care Unit | HTA looking at charting use of EMR, requirements of tasks not decision making | Not about decision making |
| Van Oldenrijk (2008) | Time-Action Analysis (TAA) of the Surgical Technique Implanting the Collum Femoris Preserving (CFP) Hip Arthroplasty. TAASTIC  trial Identifying pitfalls during the learning curve of surgeons participating in a subsequent randomized controlled trial (An  observational study) | No of cases to reach proficiency - protocol | Not about decision making |
| Yadav (2015) | Designing Real-time Decision Support for  Trauma Resuscitations | Evaluation of clinical decision support tool through simulation in traumatic brain injury | Simulation setting |
| Yagahara (2015) | Proposal for Bottom-Up Hierarchical Task Analysis: Application to the Mammography Examination Process | Unable to retrieve copy | Unable to retrieve copy |
| Yagahara (2013) | Constructing Mammography Examination Process Ontology Using Affinity Diagram and Hierarchical Task Analysis | Not decision making, description of tasks and how they fit together | Not about decision making |
| Yagahara (2018) | Construction of mammographic examination process ontology using  bottom–up hierarchical task analysis | Description of flow of mammography procedure | Not about decision making |
| Zevin (2013) | Development, Feasibility, Validity, and Reliability of  a Scale for Objective Assessment of Operative  Performance in Laparoscopic Gastric Bypass Surgery | HTA to define tasks in gastric bypass, develop assessment of skills tool, tested tool | Not about decision making |
