# Supplementary material for: The use of cognitive task analysis in clinical and health services research — a systematic review
Source: Pilot Feasibility Stud. 2022 Mar 8;8:57. doi: 10.1186/s40814-022-01002-6 (PMC8903544; doi:10.1186/s40814-022-01002-6)
Supplement: Supplementary file 2 — Additional file 2: Supplementary Table 2. Data extraction for eligible studies. [file 40814_2022_1002_MOESM2_ESM.pdf]

Supplementary Table 2 - Data extraction for eligible studies

Name (Date); Location	Title	Setting	Setting compiled	Study aims/objectives	Grouped aims/objectives	Clinical specialty	CTA Methods (2.2)	Interviews	Self-reports	Observation	Automated capture	Other	Notes	Data targets	Notes	Difficulty	Notes	Generality	Notes	Textual descriptions	Tables, Graphs, Illustrations	Qualitative models, e.g. Flowcharts	Simulation, numerical, and symbolic models	Notes
Adermann (2014); Germany	Development and validation of an artificial wetlab training system for the lumbar discectomy	Secondary care, surgery, neurosurgery, trauma and orthopaedics	Hospital	Conception and validation of a high-fidelity training model for spinal surgery, based on synthetic materials, able to mimic the optic and haptic characteristics of a real patient.	Development of training model/educational framework	Neurosurgery	Cognitive Task Analysis Interviews	Yes	No	Yes	No	No		In the Present	And interviewed on these, so also In the Past	Routine/Typical Events		Job/Task		Yes	Yes	No	No	CTA observations and interviews summarised in narrative textual description. Graphs and illustrations relate to development of simulator from the CTA information.
Ahluwalia (2018); USA	Use of Cognitive Task Analysis to Understand Decision Making for Management of Blunt Abdominal Trauma in Children	Secondary care, paediatrics	Hospital	To use cognitive task analysis to outline expert decision making process for the management of paediatric blunt abdominal injury.	Understand expert decision making for management of a particular clinical scenario	Paediatrics	Critical Decision Method (doesn't state this, but description fits) -	Yes	No	No	No	Yes	Survey and focus groups	In the Present		Routine/Typical Events		Job/Task		Yes	No	No	No	N/A
Barber (2019); Canada	Qualitative study to elicit patients' and primary care physicians' perspectives on the use of a self-management mobile health application for knee osteoarthritis	Primary care	Community/primary care	To look at patient and physician perspectives of knee osteoarthritis, management and whether they would use a self-management app.	Understand management of a procedure to investigate whether a support tool/application is warranted	Primary Care	Critical Decision Method for clinician interviews; PaCER approach for patient interviews	Yes	No	No	No	No		In the Past		Routine/Typical Events		Incident/Event		Yes	No	No	No	N/A
Barber (year unknown); Canada	Using cognitive task analysis to understand how minds work in health care	Primary care	Community/primary care	To use CTA methods to assess the following hypotheses and aims: "over ordering of MRIs is caused by a physician knowledge gap and an education campaign is needed", "create a risk calculator tool for physicians to use with patients who have knee osteoarthritis", and "a standard approach to implementing the Patient Medical Home wil work for all practices in Alberta".	Understand expert decision making for management of a particular clinical scenario; Understand a procedure with the aim of optimisation	Primary Care	Critical Decision Method; Team Knowledge Audit	Yes	No	No	No	No				Routine/Typical Events				Yes	Yes	No	No	N/A
Bartholio (2011); USA	The use of cognitive task analysis to investigate how many experts must be interviewed to acquire the critical information needed to perform a central venous catheter placement	Secondary care, large US medical centres	Hospital	To establish a recommended number of experts one would need to conduct CTA interviews with in order to capture their expertise to develop a gold standard protocol to conduct a medical procedure.	Determine the number of experts needed to develop gold standard protocol for a procedure	Trauma, Anaesthetics	Cognitive Task Analysis Interviews	Yes	No	No	No	Yes	Participant review of protocol generated from interview	In the Present	Theoretical tasks for subject of interview	Routine/Typical Events		Job/Task		Yes	Yes	No	No	Scoring of knowledge acquired from each expert on actions, decisions, objectives etc
Baxter (2005); UK	Using cognitive task analysis to facilitate the integration of decision support systems into the neonatal intensive care unit	Secondary care, NICU	Hospital	To understand the context of the NICU in order to identify how the FLORENCE system should be designed to fit in with local work practices.	Understand management of a procedure to investigate whether a support tool/application is warranted	Neonatal Intensive Care	Critical Decision Method, Field Observations	Yes	No	Yes	No	No		In the Past	CDM interviews are in the past; observations in the present.	Routine/Typical Events		Incident/Event		Yes	Yes	Yes	No	Workflow model
Boehler (2016); USA	Do surgeons and gastroenterologists describe endoscopic retrograde cholangiopancreatography differently? A qualitative study	Secondary care	Hospital	To use CTA to explore approaches by both surgeons and gastroenterologists to teaching ERCP to fellows, and to try and explain differences in opinion demonstrated in training standards.	Create framework to compare variability in a procedure	Gastroenterology; Surgery	Cognitive Task Analysis Interviews	Yes	No	No	No	No		In the Present		Routine/Typical Events		Job/Task		Yes	Yes	No	No	N/A
Boockvar (2011); USA	Medication Reconciliation: Barriers and Facilitators from the Perspectives	Tertiary care, Veterans Affairs medical centre	Hospital	To investigate factors influencing physician and pharmacist performance of medication reconciliation, using a computerised medical record and reconciliation tool. Aim to inform an organisation approach to implementation.	Understand expert decision making for management of a particular clinical scenario	Medicine; Pharmacy	Cognitive Task Analysis Interviews	Yes	No	No	No	Yes	Focus group interviews; think aloud exercise	In the Present		Routine/Typical Events		Job/Task		Yes	Yes	Yes	No	N/A
Canillas (2010); USA	The use of cognitive task analysis for identifying the critical information omitted when experts describe surgical procedures	Secondary care, Medical Centre	Hospital	To build on previous research suggesting experts omit critical information when describing procedures. To examine what knowledge experts use, and may omit when describing central venous catheter placement procedure. To look at whether the amount of prior knowledge influences the amount of knowledge omitted when experts describe the procedure.	Investigate information omission when describing/teaching a procedure	Surgery; Anaesthesia; Critical Care	Cognitive Task Analysis Interviews; Task Knowledge Structures	Yes	No	No	No	No		In the Present		Routine/Typical Events		Job/Task		Yes	Yes	No	No	N/A
Cannon-Bowers (2013); USA	Using cognitive task analysis to develop simulation-based training for medical tasks	Military training command	Other	To use CTA to identify task cues with two critical combat medical procedures. To create a set of standards to compare simulations with, allowing potential effectiveness for use in training. To investigate likely trainee errors to identify initial training requirements.	Development of training model/educational framework	Military Medicine	Cognitive Task Analysis Interviews	Yes	No	Yes	No	Yes	Think aloud	In the Present		Routine/Typical Events		Job/Task		Yes	Yes	No	No	N/A
Canopy (2015); USA	Interdisciplinary cognitive task analysis: a strategy to develop a comprehensive endoscopic retrograde cholangiopancreatography protocol for use in fellowship training	3 Tertiary academic medical centres, 1 community hospital	Hospital	To use CTA to develop an outline of procedural steps and decision points when performing and teaching endoscopic retrograde cholangiopancreatography.	To break down steps in a task to understand decision points	Gastroenterology; Surgery	Cognitive Task Analysis Interviews	Yes	No	No	No	Yes	Expert review of protocols generated from interview to create gold standard	In the Present		Routine/Typical Events		Job/Task		Yes	Yes	No	No	Procedure protocols
Chan (2017); USA	Failure to flow: an exploration of learning and teaching in busy, multi-patient environments using an interpretive description method	3 Teaching Hospitals	Hospital	To understand how emergency physicians and residents experience busy emergency department environments, and to understand how these situations affect their teaching and learning experiences.	Understand expert decision making for management of a particular clinical scenario	Emergency Medicine	Cognitive Task Analysis Interviews	Yes	No	No	No	No		In the Present		Challenging Events		Abstract/General		Yes	Yes	No	No	N/A

Chellali (2014); USA	Toward scar-free surgery: an analysis of the increasing complexity from laparoscopic surgery to NOTES	Secondary care	Hospital	To compare transvaginal NOTES cholecystectomy to laparoscopic cholecystectomy.	To compare two procedures	Surgery	Hierarchical Task Analysis; Timeline Analysis	Yes	No	Yes	No	No		In the Present		Routine/Typical Events		Job/Task		Yes	Yes	Yes	No	Hierarchical task decomposition tree
Christensen (2005); USA	Opening the black box: cognitive strategies in family practice	Family practice clinics	Community/primary care	To investigate and characterise cognitive strategies used by family physicians to structure decision tasks when seeing outpatients.	Understand expert decision making for management of a particular clinical scenario	Family Medicine	Task Diagram Construction	Yes	No	No	No	No		In the Past		Routine/Typical Events		Abstract/General		Yes	Yes	Yes	No	CTA task diagram
Cioffi (2010); Australia	The decision to suture after childbirth: cues, related factors, knowledge and experience used by midwives	Birth units	Other	To explore cues and factors when making clinical decisions on whether or not to suture perineal and associated trauma following childbirth, and to identify the knowledge acquired to support this decision making.	Understand expert decision making for management of a particular clinical scenario	Obstetrics	Critical Decision Method	Yes	No	No	No	No		In the Past		Routine/Typical Events		Incident/Event	5 cases per participant, 2 when decided to suture, 2 when not, and 1 when decided to suture and then changed to not suturing	Yes	No	No	No	N/A
Clark (2012); USA	The use of cognitive task analysis to improve instructional descriptions of procedures	Medical school, large urban research university; army and navy surgeons	University/medical school	To compare percentage of omitted knowledge by experts when describing femoral artery shunt procedure. To investigate whether task simulation increased accuracy of descriptions on recall.	Investigate information omission when describing/teaching a procedure	Trauma surgery	Cognitive Task Analysis Interviews	Yes	No	No	No	Yes	Description whilst simulating procedure	In the Past	Recall of a procedure	Routine/Typical Events		Job/Task		Yes	Yes	Yes	No	Protocol analysis
Corbett (2018); Ireland	Identifying and reducing risks in functional endoscopic sinus surgery through a hierarchical task analysis	University Hospital, secondary care	University/medical school	To develop a hierarchical task analysis for FESS procedure and analyse identified errors using SHERPA.	To break down steps in a task to understand decision points; Error identification or reduction	Surgery	Hierarchical Task Analysis; Timeline Analysis	Yes	No	Yes	No	Yes	Task breakdown, SHERPA	In the Past	Steps in a procedure	Routine/Typical Events		Job/Task		Yes	Yes	No	No	Hierarchical task analysis
Craig (2012); USA	Using cognitive task analysis to identify critical decisions in the laparoscopic environment	Secondary care	Hospital	To use CTA to identify decisions, cues, strategies and novice traps relevant in laparoscopic surgery.	Understand expert decision making for management of a particular clinical scenario	Surgery	Critical Decision Method; Knowledge Audit	Yes	No	No	No	No		In the Past		Routine/Typical Events		Abstract/General		Yes	Yes	No	No	Decision requirements table; expert checking
Crandall (1993); USA	Critical Decision Method: A technique for eliciting concrete assessment indicators from the intuition of NICU nurses	Secondary care; NICU, regional referral centre	Hospital	To use CDM to extract key clinical judgement components from nurses. To develop a set of instructional material in early indicators of sepsis in low birth weight infants for novice NICU nurses.	Development of training model/educational framework	Neonatal Intensive Care	Critical Decision Method	Yes	No	No	No	Yes	Evaluation questionnaire	In the Past		Routine/Typical Events	Participants asked to select a case where they believed they had made a difference to the patient's outcome. Second study - asked to recall a case with correct suspicion of sepsis, one incorrect suspicion and one where sepsis was not detected but was present.	Incident/Event		Yes	Yes	No	No	Guide to early sepsis detection
Crispen (2010); USA	Identifying the point of diminishing marginal utility for cognitive task analysis surgical subject matter expert interviews	Private, urban medical school in US	University/medical school	To investigate how much information about open cricothyrotomy one expert can provide when compared to gold standard summary based in interviews with 6 experts. To look at how much critical information is gained from each additional CTA interview.	Methodological (saturation)	Surgery	Critical Decision Method (doesn't state this, but description fits)	Yes	No	No	No	Yes	Development of a gold standard, and comparison of each expert interview against this gold standard	In the Past	Recalling steps in a procedure	Routine/Typical Events		Job/Task		Yes	Yes	No	No	Protocol analysis
Crowley (2001); USA	Development of visual diagnostic expertise in pathology: an information-processing study	University medical centre system	University/medical school	To use information-processing and cognitive science methodologies to compare visual diagnostic processes of novices and experts. To gain insights for developing an intelligent pathology tutoring system.	Development of training model/educational framework	Pathology	Think aloud	No	No	No	Yes	Yes	Think aloud protocols	In the Present		Routine/Typical Events		Job/Task		Yes	Yes	No	Yes	**Task timeline** depicting latencies and event counts. Recall, evidence, hypothesism relationship; Evaluate, certainty, hypothesis;
Custer (2012); USA	A qualitative study of expert and team cognition on complex patients in the paediatric intensive care unit	Secondary care; PICU within academic children's centre	Hospital	To define the processes and limits of expert and team cognition, to enable support systems that improve team dynamics, outcomes and educational models	Development of training model/educational framework	Paediatric Intensive Care	Cognitive Task Analysis Interviews	Yes	No	No	No	No		In the Present		Challenging Events	"Complex"	Incident/Event		Yes	Yes	No	No	Grounded theory; Mental model breakdown
Demirel (2019); USA	Design of virtual interactive simulations for surgical training	Secondary care	Hospital	To develop a VR based arthroscopic rotator cuff tear diagnosis and repair surgical simulator, which could look at the skill level of the surgeon. To compare performance between novice and expert surgeons for this procedure.	Simulator development; Compare differences between novices and experts	Surgery	Hierarchical Task Analysis; video timing analysis	No	No	Yes	No	Yes	Questionnaire on details of the procedure	In the Past		Routine/Typical Events		Job/Task		Yes	Yes	Yes	No	Hierarchical Task Analysis Tree
Diwadkar (2012); USA	Understanding the critical components of performing vaginal hysterectomy with cognitive task analysis	Womens Health Institute	Hospital	To use CTA to identify critical steps in vaginal hysterectomy and the decisions required to devise a more effective way of teaching and assessing this procedure.	To understand critical steps in a procedure to provide effective teaching and procedure assessment	Gynaecology	Critical Decision Method (doesn't state this, but description fits)	Yes	No	Yes	No	No		In the Past		Routine/Typical Events		Job/Task		Yes	No	Yes	No	Decision tree
Fackler (2009); USA	Critical care physician cognitive task analysis: an exploratory study	Intensive care units within major university teaching hospitals	Hospital	To investigate whether CTA could be used for physician-team task analysis to guide restructuring and/or task reallocation.	Understand a procedure with the aim of optimisation	Critical care	Critical Decision Method	Yes	No	Yes	No	No		In the Present		Routine/Typical Events		Incident/Event		Yes	No	No	No	N/A
Fioratou (2016); UK	Beyond monitors: distributed situation awareness in anaesthesia management	Obstetric units in NHS hospitals in Scotland	Hospital	To investigate how DSA is used in decision making in major obstetric haemorrhage. How do anaesthetists understand, and anticipate when faced with challenging cases.	Understand expert decision making for management of a particular clinical scenario	Obstetric Anaesthesia	Distributed situation awareness; Critical Decision Method	Yes	No	No	No	No		In the Past		Challenging Events		Incident/Event		Yes	Yes	No	No	N/A
Gawande (2003); USA	Analysis of errors reported by surgeons at three teaching hospitals	US Teaching hospital	Hospital	To see if a confidential interview approach is a feasible and useful method to investigate reports on adverse events resulting from errors in surgical care.	Error identification or reduction	Surgery	Cognitive Task Analysis Interviews; Incident Analysis	Yes	No	No	No	No	Identification of adverse event reports submitted to M&M meetings	In the Past		Routine/Typical Events	Adverse events, but not noted to be anything challenging within the particular events selected	Incident/Event		Yes	Yes	No	No	N/A
Gazarian (2015); USA	A description of nurses' decision making in managing electrocardiographic monitor alarms	Academic medical centre in NE USA	Hospital	To describe cues and factors nurses use in decision making when responding to clinical alarms. Overall aim is to create an evidence-based education to teach nurses to correctly identify and interrupt clinical alarms.	To understand critical steps in a procedure to provide effective teaching and procedure assessment	General Medicine	Critical Decision Method	Yes	No	Yes	No	No	Observations to identify cases, for discussion in interview	In the Past	In the present during the observation period; in the past for the interview	Routine/Typical Events		Incident/Event		Yes	Yes	No	No	N/A

Gazarian (2010); USA	Nurse decision making in the prearrest period	Academic medical centre in NE USA, considered national leader in quality improvement and patient safety initiatives	Hospital	To describe the cues and factors influencing the decision making used by nurses when identifying and interrupting a potential cardiopulmonary arrest.	Understand expert decision making for management of a particular clinical scenario	Critical care	Critical Decision Method	Yes	No	No	No	No		In the Past		Routine/Typical Events	Cases identified and then nurses involved in their care were invited to participate. 4 typical, 9 complex cases	Incident/Event		Yes	No	No	No	N/A
Geis (2016); USA	Leveraging the Critical Decision Method to develop simulation-based training for early recognition of sepsis	Urban, academic quaternary care paediatric hospital	Hospital	To explore differences in cue recognition and utilisation between physicians with different levels of expertise. The wider goal is to develop learning strategies to improve recognition and treatment of sepsis.	Development of training model/educational framework; Compare differences between novices and experts	Paediatrics (ED, PICU, NICU)	Critical Decision Method	Yes	No	No	No	No		In the Past	Tried to avoid participants thinking of incidents prior to the interview - did not tell them study was about sepsis recognition	Routine/Typical Events	Doesn't really specify, participants asked to recall an incident where sepsis was suspected or later discovered	Incident/Event		Yes	Yes	No	No	Uses timelines as part of the CTA Method, but this isn't the same as results display
Grunwald (2004); USA	Using cognitive task analysis to facilitate collaboration in development of simulator to accelerate surgical training	Research Centre	University/medical school	To describe a framework which can be used to look at a surgical procedure and develop technology to complement educational and cognitive goals of the user.	Development of training model/educational framework	Surgery	Cognitive Task Analysis Interviews; Task Knowledge Structures	Yes	No	No	No	Yes	Develop task analysis outline from expert interviews, to review over multiple iterations	In the Present		Routine/Typical Events		Abstract/Genera l		Yes	No	Yes	No	Workflow model
Harencarova (2017); Slovakia	Managing uncertainty in paramedics' decision making	ERS ambulance crews (non-physician staffed ambulances)	Pre-hospital	To identify the uncertainties paramedics face and strategies used to manage uncertainty at different times within an incident. To describe what uncertainty in this domain means.	Understand expert decision making for management of a particular clinical scenario	Paramedic Medicine	Critical Decision Method	Yes	No	No	No	No		In the Past		Rare Events & Anomalies		Incident/Event		Yes	Yes	No	No	Decision analysis table
Harencarova (2015); Slovakia	Structured analysis of critical decision method data - emergency medicine case study	Pre-hospital	Pre-hospital	To identify types of uncertainty and strategies used to manage uncertainty in non-routine situations in paramedics	Understand expert decision making for management of a particular clinical scenario	Paramedic Medicine	Critical Decision Method	Yes	No	No	No	No		In the Past		Rare Events & Anomalies	Non routine	Incident/Event		Yes	Yes	Yes	No	Decision analysis table; Decision chart
Hashimoto (2019); USA	Surgical procedural map scoring for decision making in laparoscopic cholecystectomy	Academic institution	University/medical school	To investigate surgeons' decision making using CTA to capture procedural maps. To compare two scoring systems for procedural maps in their ability to identify deicision making differences between attending and resident surgeons.	Create framework to compare variability in a procedure; Compare differences between novices and experts	Surgery	Cognitive Task Analysis interviews	Yes	No	No	No	No		In the Present		Routine/Typical Events		Job/Task		Yes	Yes	Yes	No	Procedural map/consensus procedural map
Hou (2017); USA	Assessment of gaps in care and the development of a care pathway for anemia in patients with inflammatory bowel diseases	Healthcare providers	Hospital	To identify current practices and perceptions of anemia and develop a pathway for screening, management and follow up of anemia among patients with IBD	Understand expert decision making for management of a particular clinical scenario	Gastroenterology	Knowledge Analysis	No	Yes	No	No	Yes	Survey, focus group	In the Present		Routine/Typical Events		Job/Task		Yes	Yes	Yes	No	Workflow model
Islam (2015); USA	Understanding complex clinical reasoning in infectious diseases for improving clinical decision support design	Veterans Medical Center, and a University hospital	Hospital	To identify decision complexity and strategies to inform design of health information technology.	Understand expert decision making for management of a particular clinical scenario	Infectious Diseases	Critical Decision Method	Yes	No	No	No	No		In the Past	Recent case	Challenging Events		Incident/Event		Yes	Yes	Yes	No	Content analysis
Islam (2014); USA	Heuristics in managing complex clinical decision tasks in experts' decision making	Veterans Medical Center, and a University hospital	Hospital	To investigate different heuristics clinical experts adopt for making complex clinical decisions.	Understand expert decision making for management of a particular clinical scenario	Infectious Diseases, Geriatric Medicine & Palliative Care	Critical Decision Method	Yes	No	No	No	No	Pilot work	In the Past	Recent case	Challenging Events		Incident/Event		Yes	Yes	Yes	No	N/A
Jacklin (2008); UK	Mapping surgical practice decision making: an interview study to evaluate decisions in surgical care	A teaching hospital and a district general hospital in the UK	Hospital	To map process of care through decision making by identifying decisions in symptomatic gallstone disease. To test whether cognitive surgical skill of decision making could be deconstructed into an accessible format for surgical trainees.	Understand expert decision making for management of a particular clinical scenario	Surgery	Cognitive Task Analysis Interviews	Yes	No	No	No	No		In the Future		Routine/Typical Events		Job/Task		Yes	Yes	Yes	No	Process mapping
Johnson (2006); UK	Physical and cognitive task analysis in interventional radiology	Secondary care	Hospital	To use CTA to develop protocols covering processes required to to successfully complete a task within interventional radiology, potentially for use in training.	Development of training model/educational framework	Interventional Radiology	Cognitive Task Analysis Interviews; Task Breakdown	Yes	No	Yes	No	No		In the Present		Routine/Typical Events		Job/Task		Yes	Yes	No	No	Protocol development
Khandan (2017); Iran	SHERPA technique as an approach to healthcare error management and patient safety improvement: a case study among nurses	Teaching hospital	Hospital	To assess the types and causes of errors among nurses, and offer solutions to manage nurse tasks and reduce the incidence of medical errors.	Error identification or reduction	Women's Infectious Diseases	SHERPA; Hierarchical Task Analysis	Yes	No	Yes	No	Yes	SHERPA worksheets to classify tasks	In the Present		Routine/Typical Events		Abstract/Genera l		Yes	Yes	No	No	Hierarchical task analysis; task breakdown
Kim (2013); USA	The use of cognitive task analysis to determine surgical expert's awareness of critical decisions required for a surgical procedure	School of Medicine within Medical Center	University/medical school	To investigate surgeons ability to recall indications and contraindications for CVC placement.	Understand expert decision making for management of a particular clinical scenario	Surgery	Cognitive Task Analysis Interviews	Yes	No	No	No	Yes	Development of gold standard protocol to compare each interview with	In the Present		Routine/Typical Events		Job/Task	to examine specifically the omission of decisions by medical experts when describing central venous catheter placement	Yes	Yes	No	No	Protocol analysis
Knight (2018); France	Development of an objective assessment tool for total laparoscopic hysterectomy: a Delphi method among experts and evaluation on a virtual reality simulator	Teaching hospital	Hospital	To develop an objective scale for assessing technical skills for laparoscopic hysterectomy and to show feasibility and validity in a VR setting.	Development of training model/educational framework	Gynaecology	Hierarchical Task Analysis; Delphi method for consensus of H-OSATS scale	No	No	Yes	No	No		In the Present		Routine/Typical Events		Job/Task		Yes	Yes	No	Yes	H-OSATS scale
Lajoie (1998); Canada	Cognitive Tools for assessment and learning in a high information flow environment	Large metropolitan university teaching hospital	Hospital	To discuss the scope of curriculum for the types of problems and learning objectives that need to be addressed in the SICU	Development of training model/educational framework	SICU	Cognitive Task Analysis Interviews; Think aloud	Yes	No	No	No	Yes	Think aloud descriptions	In the Future	Given a scenario	Routine/Typical Events	Given a scenario	Incident/Event	Hypothetical	Yes	Yes	Yes	No	N/A
Madani (2017); USA	What are the principles that guide behaviours in the operating room?	Universities	University/medical school	To map process of expert surgeons through defining principles guiding their decisions and to develop a framework reflecting intraoperative performance.	Understand expert decision making for management of a particular clinical scenario	Surgery	Cognitive Task Analysis interviews; grounded theory; hierarchical task analysis	Yes	No	Yes	No	Yes	Think aloud descriptions	In the Present		Routine/Typical Events		Job/Task		Yes	Yes	Yes	No	Conceptual framework
Melnick (2015); USA	Understanding overuse of computed tomography for minor head injury in the emergency department: a triangulated qualitative study	Urban, academic trauma center ED	Hospital	To understand the challenges in implementing appropriate CT use to inform future strategies in optimisation of CT use.	Understand a procedure with the aim of optimisation	Trauma medicine	Critical Decision Method	Yes	No	Yes	No	Yes	Focus groups with patients and care providers	In the Present	Observed, then interviewed about the case	Routine/Typical Events		Incident/Event		Yes	Yes	No	No	N/A

Militello (1995); USA	Patient assessment skills: assessing early cues of necrotising enterocolitis	Regional referral centres, NICU	Hospital	To look at the feasibility of CTA methods in extracting expert nurse knowledge of assessment of risk of necrotising enterocolitis.	Understand expert decision making for management of a particular clinical scenario	Neonatal Intensive Care	Critical Decision Method	Yes	No	No	No	No		In the Past		Routine/Typical Events	A case where nurse suspected the patient was developing necrotising enterocolitis and that turned out to be correct; a case where suspicion of NEC was incorrect; a case where NEC developed but wasn't suspected	Incident/Event	A case where nurse suspected the patient was developing necrotising enterocolitis and that turned out to be correct; a case where suspicion of NEC was incorrect; a case where NEC developed but wasn't suspected	Yes	Yes	No	No	Strategies analysis; framework development
Militello (1995)	A cognitive task analysis of NICU nurses' patient assessment skills	DUPLICATE OF ABOVE				Neonatal Intensive Care																		
Militello (2018); USA	Understanding how primary care clinicians make sense of chronic pain	Primary care	Community/Primary care	To use the data frame theory of sensemaking to look at how primary care clinicians in the USA manage patients with chronic non-cancer pain.	Understand expert decision making for management of a particular clinical scenario	Primary care	Critical Decision Method	Yes	No	No	No	No	Patient cases identified and patients agreed to be subject of interview	In the Past		Routine/Typical Events		Job/Task		Yes	Yes	Yes	No	N/A
O'Sullivan (2011); Ireland	Proactive error analysis of ultrasound-guided axillary brachial plexus block performance	Tertiary referral, university teaching hospital	Hospital	To use CTA methods to determine a description of tasks when performing ultrasound guided brachial plexus blockade, to identify elements with the potential for human error.	Error identification or reduction	Anaesthesia	Hierarchical Task Analysis; Cognitive Task Analysis Interviews; SHERPA	Yes	No	No	No	Yes	Generated HTA of procedure using educational resources. Questionnaire for experts to review list of credible errors identified through SHERPA	In the Present		Routine/Typical Events		Job/Task		Yes	Yes	Yes	No	Hierarchical Task Analysis; Protocol analysis
Patterson (2016)	Leveraging the Critical Decision Method to develop simulation-based training for early recognition of sepsis	DUPLICATE OF GEIS																						
Pauley (2013); UK	Intraoperative decision making by ophthalmic surgeons	Secondary care	Hospital	To look at decision making and risk management processes in ophthalmic surgery	Understand expert decision making for management of a particular clinical scenario	Ophthalmic surgery	Critical Decision Method	Yes	No	No	No	No		In the Past	Up to 2 years before interview took place	Challenging Events		Incident/Event		Yes	Yes	No	No	N/A
Pauley (2011); UK	Surgeons' intraoperative decision making and risk management	3 teaching hospitals in Scotland	Hospital	To investigate types of decision making processes used by surgeons and whether these differ depending on situational factors.	Understand expert decision making for management of a particular clinical scenario	Surgery	Critical Decision Method	Yes	No	No	No	No		In the Past	Up to 12 years before interview took place	Challenging Events		Incident/Event		Yes	Yes	No	No	N/A
Pryor (2006); Australia	What do nurses do in response to their predictions of aggression?	Brain injury rehabilitation units	Other	To identify responses that nurses make to predictions of aggression in acquired brain injury.	Understand expert decision making for management of a particular clinical scenario	Neuro-rehabilitation	Critical Decision Method	Yes	No	No	No	No		In the Past		Routine/Typical Events		Incident/Event		Yes	Yes	No	No	N/A
Pryor (2005); Australia	What cues do nurses use to predict aggression in people with acquired brain injury?	Brain injury rehabilitation units	Other	To identify the cues used in predicting aggression in people with acquired brain injury.	Understand expert decision making for management of a particular clinical scenario	Neuro-rehabilitation	Critical Decision Method	Yes	No	No	No	No		In the Past		Routine/Typical Events		Incident/Event		Yes	No	No	No	N/A
Pugh (2011); USA	Intraoperative decision making: more than meets the eye	Secondary care	Hospital	To understand the complexity of intraoperative decision making in surgery	Understand expert decision making for management of a particular clinical scenario	Surgery	Concepts, Processes and principles approach	Yes	No	No	No	No		In the Present		Routine/Typical Events		Job/Task		Yes	Yes	Yes	No	Decision trees; decision charts
Raduma-Tomas (2012); UK	The importance of preparation for doctors' handovers in an acute medical assessment unit: a hierarchical task analysis	Acute Medical Assessment Unit in a teaching hospital	Hospital	To describe the ideal doctors handovers in acute medical assessment unit. To compare actual handovers against the ideal.	Create framework to compare variability in a procedure	Acute Medical Assessment unit	Hierarchical Task Analysis	Yes	No	Yes	No	Yes	Focus group	In the Present		Routine/Typical Events		Job/Task		Yes	No	Yes	No	Hierarchical Task Analysis
Rimstad (2015); Norway	A retrospective observational study of medical incident command and decision making in the 2011 Oslo bombing	Pre-hospital	Pre-hospital	To describe critical decisions ambulance commander and medical commander make in a mass casualty incident response, and to understand what underlies decision making	Understand expert decision making for management of a particular clinical scenario	Pre-hospital/Emergency	Critical Decision Method; Cognitive Task Analysis Interviews	Yes	No	Yes	No	No	Recordings of radio communications	In the Past		Rare Events & Anomalies		Incident/Event		Yes	Yes	No	No	Systematic Text condensation and timelines
Sarker (2008); UK	Decision making in laparoscopic surgery: a prospective, independent and blinded analysis	Secondary care (2 district general hospitals, 2 teaching hospitals)	Hospital	To describe factors influencing decision making in laparoscopic surgery	Understand expert decision making for management of a particular clinical scenario	Surgery	Hierarchical Task Analysis	No	No	Yes	No	No		In the Present		Routine/Typical Events		Job/Task		Yes	Yes	Yes	No	Decision making model; Hierarchical Task Analysis
Schnittker (2017,2018); Australia	Human factors enablers and barriers for successful airway management - an in-depth interview study	Secondary care	Hospital	2017: To look at cognitive pathways underlying decisions in challenging airway management situations.; 2018: To provide guidance on design of decision support by understanding how human factors affect airway management.	2017: Understand expert decision making for management of a particular clinical scenario; 2018: Understand management of a procedure to investigate whether a support tool/application is warranted	Anaesthesia	Critical Decision Method	Yes	No	No	No	No		In the Past		Challenging Events		Incident/Event		Yes	Yes	Yes	No	N/A
Schreiner (2009); USA	Facing the diabetes epidemic: expertise among diabetes educators	Secondary care	Hospital	To determine how expert diabetes educators practice, make clinical decisions, and sustain expertise	Understand expert decision making for management of a particular clinical scenario	Diabetes Education	Critical Decision Method	Yes	Yes	No	No	Yes	Photo elicitation, limited information task, participant diary, survey of self learning, Think aloud	In the Past		Routine/Typical Events		Abstract/General		Yes	Yes	Yes	No	Human Performance Improvement model; Thematic network
Schubert (2013); USA	Characterising novice-expert differences in macrocognition: an exploratory study of cognitive work in the emergency department	Tertiary medical centre	Hospital	To investigate the differences in macrocognition between experts and novices in emergency medical work.	Compare differences between novices and experts	Emergency Medicine	Critical Decision Method	Yes	No	No	No	No		In the Past		Challenging Events		Incident/Event		Yes	Yes	Yes	No	N/A
Sedlar (2017); Slovakia	Situation assessment and decision making strategies of emergency medical services physicians in routine and non-routine situations.	Emergency Medicine	Hospital	To identify which situation assessment and decision making strategies from NDM perspective emergency physicians use in real world situations.	Understand expert decision making for management of a particular clinical scenario	Emergency Medicine	Critical Decision Method	Yes	No	No	No	No		In the Past		Routine/Typical Events	And non-routine	Incident/Event		Yes	Yes	No	No	N/A
Segall (2013); USA	A cognitive modeling approach to decision support tool design for anaesthesia provider crisis management	University Human Simulation and Patient Safety Centre	University/Medical school	To use HTA techniques to provide a human centred approach to development of a crisis management decision support tool.	Understand management of a procedure to investigate whether a support tool/application is warranted	Anaesthesia	Hierarchical Task Analysis; Cognitive Task Analysis Interviews	Yes	No	No	No	No		In the Present		Routine/Typical Events		Job/Task		Yes	Yes	Yes	No	Hierarchical Task Analysis; GOMS model; Cogntive Function Model

Sitterding (2014); USA	Situation awareness and interruption handling during medication administration	Secondary care	Hospital	To describe situation awareness during medication administration, and strategies for handling interruptions.	Understand expert decision making for management of a particular clinical scenario	Critical care/Medicine/Surgery	Critical Decision Method	Yes	No	Yes	No	No		In the Past		Routine/Typical Events		Job/Task		Yes	Yes	No	No	N/A
Slagle (2005); USA	The construction and validity assessment of a decision making and performance measure derived from cognitive task analysis	University residency program	University/Medical school	To use cognitive task analysis to understand decision making and performance of high risk tasks in extubation	Understand expert decision making for management of a particular clinical scenario	Anaesthesia	Critical Decision Method	Yes	No	No	No	No		In the Past		Challenging Events		Incident/Event		Yes	Yes	No	Yes	N/A
Smink (2012); USA	Utilisation of a cognitive task analysis for laparoscopic appendectomy to identify differentiated intraoperative teaching objectives.	Secondary care	Hospital	To use CTA to describe key steps and decision points in laparoscopic appendectomy. To use this information to identify teaching objectives.	To understand critical steps in a procedure to provide effective teaching and procedure assessment	Surgery	Critical Decision Method (not stated but description fits)	Yes	No	No	No	Yes	Consensus on cognitive demands table	In the Past		Challenging Events		Incident/Event		Yes	Yes	No	No	Cognitive Demands Table
Smith (2018); USA	Incorporating guideline adherence and practice implementation issues into the design of decision support for beta-blocker titration for heart failure	Veterans Health Administration	Hospital	To develop a design for a clinical decision support tool, using CTA methods.	Understand management of a procedure to investigate whether a support tool/application is warranted	Primary care	Critical Decision Method	Yes	No	No	No	No		In the Past		Routine/Typical Events		Incident/Event		Yes	Yes	No	No	N/A
Sowb (1998); USA	Cognitive modeling of intraoperative critical events	Secondary care	Hospital	To investigate sources of difficulty and role of equipment in supporting clinical decisions during intraoperative ventilation related events	Understand expert decision making for management of a particular clinical scenario	Anaesthesia	Cognitive Task Analysis Interviews	Yes	No	No	No	No		In the Past		Routine/Typical Events		Incident/Event		Yes	No	Yes	No	Functional problem solving model; Abstraction hierarchy
Sullivan (2008); USA	Assessing the teaching of procedural skills: can cognitive task analysis add to our traditional teaching methods?	Secondary care	Hospital	To identify omitted steps by surgeons when teaching colonoscopy.	Investigate information omission when describing/teaching a procedure	Surgery	Think aloud; Cognitive Task Analysis Interviews	Yes	No	Yes	No	Yes	Think aloud	In the Present		Routine/Typical Events		Incident/Event		Yes	Yes	No	No	N/A
Sullivan (2014); USA	The use of cognitive task analysis to reveal the instructional limitations of experts in the teaching of procedural skills	Secondary care	Hospital	To identify the amount of clinical knowledge omitted by experts when teaching cricothyrotomy, and to identify the gaps with CTA interview protocol.	Investigate information omission when describing/teaching a procedure	Trauma surgery	Cognitive Task Analysis Interviews; Concepts, Process and Principles approach	Yes	No	Yes	No	Yes	Develop gold standard protocol from video observations	In the Present		Routine/Typical Events		Job/Task		Yes	Yes	No	No	Protocol analysis
Tolano-Leveque (2011); USA	Using Cognitive Task Analysis to determine the percentage of critical information that experts omit when describing a surgical procedure	School of Medicine	University/Medical school	To look at the percentage of critical "when" and "how" information experts omit when describing open cricothyrotomy procedure, and whether the amount of an expert's prior knowledge and experience influences the amount of knowledge omitted when describing how to perform the same procedure.	Investigate information omission when describing/teaching a procedure	Surgery	Cognitive Task Analysis Interviews; Task Knowledge Structures	Yes	No	No	No	No	Comparison of steps identified in interviews against "gold standard"	In the Past		Routine/Typical Events		Job/Task		Yes	Yes	Yes	No	CTA protocol for the procedure
Varga (2013); Netherlands	Manipulation of mental models of anatomy in interventional radiology and its consequences for design of human-computer interaction	Secondary care, Medical Centre	Hospital	To understand the cognitive and physical processes of interventional radiology in order to elicit relevant information for user interface design for human-computer interaction	Understand expert decision making for management of a particular clinical scenario	Interventional Radiology	Cognitive Task Analysis Interviews; Task Knowledge Structures	Yes	No	Yes	No	Yes	Think aloud	In the Present		Routine/Typical Events		Abstract/General	Interventional Radiology, not a specific procedure	Yes	Yes	Yes	No	Task analysis; workflow model
Wannheden (2013); Sweden	HIV and tuberculosis coinfection - a qualitative study of treatment challenges faced by care providers	University Hospital	Hospital	To understand challenges faced when treating patients coinfected with HIV and TB, and to identify opportunities for IT based support.	Understand management of a procedure to investigate whether a support tool/application is warranted	Infectious Diseases	Critical Decision Method; task diagram interview	Yes	No	No	No	No		In the Past		Challenging Events		Incident/Event		Yes	Yes	No	No	N/A
Weinger (2002); USA	Human Factors Research in Anaesthesia Patient Safety: Techniques to elucidate factors affecting clinical task performance and decision making	Secondary care	Hospital	To investigate clinical decision making in non-routine events in anaesthesia	Understand expert decision making for management of a particular clinical scenario	Anaesthesia	Task knowledge structures; cognitive task analysis interviews; behavioural task analysis	Yes	No	No	No	No		In the Past		Challenging Events	Specific notable or difficult clinical decision	Incident/Event		Yes	Yes	No	No	Cognitive map
White (2018); Canada	Getting inside the expert's head: an analysis of physician cognitive processes during trauma resuscitations	Trauma teams, secondary care	Hospital	To understand the specific cognitive processes of physicians when leading resuscitations. To look at the feasibility of using eye tracking technology to enhance traditional CTA techniques.	Understand management of a procedure to investigate whether a support tool/application is warranted	Resuscitation Medicine	Cognitive Task Analysis Interviews	Yes	No	Yes	Yes	Yes	Eye tracking glasses to observe cases, then "think aloud" whilst participants viewed the recorded videos	In the Past	Cases recorded in the present, but interviews after they occurred	Routine/Typical Events	Selection of cases, not influenced by participants	Incident/Event		Yes	Yes	Yes	No	Cognitive process map
Woolley (2013); UK	Clinical intuition in family medicine: more than first impressions	Primary care	Community/Primary care	To look at intuition and identify cognitive processes in medical decision making.	Understand expert decision making for management of a particular clinical scenario	Family Medicine	Critical Decision Method	Yes	No	No	No	No		In the Past		Routine/Typical Events	Two cases for each participant, one where intuition was correct, one where it was incorrect	Incident/Event		Yes	No	No	No	N/A
Yates (2012); USA	Integrated studies on the use of cognitive task analysis to capture surgical expertise for central venous catheter placement and open cricothyrotomy	Secondary care	Hospital	To determine the amount of knowledge experts omit when describing a procedure, how many experts are required to capture the essential procedure knowledge and to investigate the effectiveness of training when based on CTA.	Investigate information omission when describing/teaching a procedure	Surgery	Cognitive Task Analysis Interviews; protocol generation	Yes	No	No	No	Yes	Development of protocol for each interview, put together to create gold standard	In the Present		Routine/Typical Events		Job/Task		Yes	Yes	No	No	N/A
Yeh (2015); Taiwan	Using cognitive task analysis to teach laparoscopic nephrectomy for living related kidney transplantation	Secondary care	Hospital	To look at how CTA can be used to teach laparoscopic nephrectomy for living related kidney transplantation.	Development of training model/educational framework	Surgery	Task knowledge structures	No	No	Yes	No	No		In the Present		Routine/Typical Events		Job/Task		Yes	No	No	No	Videos and written documents produced from observations for use in teaching.
Yeh (2015); Taiwan	Developing a training module for living related liver transplantation by using CTA method	Secondary care	Hospital	To look at how CTA can be used to develop teaching module for living related liver transplantation.	Development of training model/educational framework	Surgery	Task knowledge structures	No	No	Yes	No	No		In the Present		Routine/Typical Events		Job/Task		Yes	No	No	No	Videos and written documents produced from observations for use in teaching.

Yeung (2017); Canada	Developing cognitive task analysis-based educational videos for basic surgical skills in plastic surgery	Urban, tertiary care teaching centre	Hospital	To describe the use of CTA to develop educational materials for plastic surgery skills.	Development of training model/educational framework	Plastic Surgery	Needs assessment survey; Task knowledge structures	Yes	No	Yes	No	No	After survey to list skills, experts delivered training, were videoed and then interviewed to deconstruct each skill	In the Present		Routine/Typical Events	Teaching particular procedures	Job/Task		Yes	Yes	No	No	Development of training videos
Yu (2014); USA	Identification of technique variations among microvascular surgeons and cases using hierarchical task analysis	University Hospital	Hospital	To create a systematic framework to compare variability in surgical technique.	Create framework to compare variability in a procedure	Surgery	Hierarchical Task Analysis	Yes	No	Yes	No	No		In the Present		Routine/Typical Events		Job/Task		Yes	Yes	No	Yes	Creation of taxonomy; Hierarchical task analysis
Zupanc (2015); Australia	A competency framework for colonoscopy training derived from cognitive task analysis techniques and expert review	Three metropolitan hospitals (Sydney, Melbourne, Brisbane)	Hospital	To supplement existing colonoscopy curriculum documents with a competency framework for training the procedure based on CTA and expert review.	Development of training model/educational framework	Endoscopy	Observation, think aloud, cognitive task analysis interviews	Yes	No	Yes	No	Yes	Think aloud	In the Present		Routine/Typical Events		Job/Task		Yes	No	Yes	No	Competency framework
